# Is Lactate an Oncometabolite? Evidence Supporting a Role for Lactate in the Regulation of Transcriptional Activity of Cancer-Related Genes in MCF7 Breast Cancer Cells

**DOI:** 10.3389/fonc.2019.01536

**Published:** 2020-01-14

**Authors:** Iñigo San-Millán, Colleen G. Julian, Christopher Matarazzo, Janel Martinez, George A. Brooks

**Affiliations:** ^1^Department of Medicine, Division of Endocrinology, Metabolism and Diabetes, University of Colorado School of Medicine, Aurora, CO, United States; ^2^Department of Human Physiology and Nutrition, University of Colorado, Colorado Springs, CO, United States; ^3^Department of Medicine, University of Colorado School of Medicine, Aurora, CO, United States; ^4^Department of Integrative Biology, University of California, Berkeley, Berkeley, CA, United States

**Keywords:** lactate, cancer, oncogenes, transcription factors, cell cycle genes

## Abstract

Lactate is a ubiquitous molecule in cancer. In this exploratory study, our aim was to test the hypothesis that lactate could function as an oncometabolite by evaluating whether lactate exposure modifies the expression of oncogenes, or genes encoding transcription factors, cell division, and cell proliferation in MCF7 cells, a human breast cancer cell line. Gene transcription was compared between MCF7 cells incubated in (a) glucose/glutamine-free media (control), (b) glucose-containing media to stimulate endogenous lactate production (replicating some of the original Warburg studies), and (c) glucose-containing media supplemented with L-lactate (10 and 20 mM). We found that both endogenous, glucose-derived lactate and exogenous, lactate supplementation significantly affected the transcription of key oncogenes (MYC, RAS, and PI3KCA), transcription factors (HIF1A and E2F1), tumor suppressors (BRCA1, BRCA2) as well as cell cycle and proliferation genes involved in breast cancer (AKT1, ATM, CCND1, CDK4, CDKN1A, CDK2B) (0.001 < *p* < 0.05 for all genes). Our findings support the hypothesis that lactate acts as an oncometabolite in MCF7 cells. Further research is necessary on other cell lines and biopsy cultures to show generality of the findings and reveal the mechanisms by which dysregulated lactate metabolism could act as an oncometabolite in carcinogenesis.

## Introduction

A role of lactate in cancer metabolism was first described almost a century ago when Otto Warburg and associates discovered that cancer cells were not only characterized by accelerated glucose consumption, but also by a marked increase in lactate production (1). They exposed tumor cells to amino acids, fatty acids, and glucose, expecting the highest rate of respiration in glucose-exposed cancer cells. Contrary to expectations, glucose brought respiration to a standstill. “*In trying to discover why this happened, it was found that lactic acid appeared in the Ringer's solution, produced by glycolysis, and that this inhibited the respiration*,” asserted Warburg (2). Warburg also found that arterial glucose uptake in tumor cells was about 47–70%, compared to 2–18% in normal tissues, and that tumor cells converted 66% of glucose uptake to lactate (3). Based on these observations, Warburg concluded that increased glycolytic activity was integral to carcinogenesis, a phenomenon subsequently termed the “Warburg Effect” (4).

In the last decade there has been a “renaissance” of cancer metabolism and the knowledge acquired has been significant. It is now known that carcinogenesis involves complex metabolic processes characterized by tumor heterogenicity, involving different metabolic activities and regulations necessary for tumor growth, survival and progression (5). Increasing body of literature implicates the involvement of lactate for carcinogenesis. Sonveaux et al. showed that lactate is a major fuel for biomass and anabolic necessities of cancer cells (6, 7).

Given the unexplained causes and consequences of the Warburg effect in cancer, recently, we articulated the “lactagenesis hypothesis” (8). According to our hypothesis, the predominant role of lactate in cancer is not only for fuel or cancer biomass purposes but also for carcinogenic signaling properties. Lactate is involved in the main biological processes that are known to drive or sustain carcinogenesis, specifically: angiogenesis, immune escape, cell migration, metastasis, and self-sufficient metabolism (8).

While lactate has been the subject of intense investigation since at least the nineteenth century, until recently it was believed that lactate was solely a byproduct of oxygen-limited, anaerobic metabolism. However, in the mid 1980's George Brooks proposed the “Lactate Shuttle Hypothesis” based on results of isotope tracer studies in rodents and humans (9–13). His studies showed for the first time that lactate production and exchange could also occur under fully aerobic conditions debunking the belief that lactate was a “waste” product of anaerobic glucose metabolism (14). Specifically to cancer cells, it is estimated that in cancer cells, lactate production accounts ~70% from aerobic glycolysis (15).

Furthermore, it is now recognized that lactate is a major energy source (16–19), the major gluconeogenic precursor (19), a signaling molecule and a “lactormone” (13) that also influences gene expression (20–23). Exogenous L-lactate exposure, for instance, has been reported to upregulate the transcriptional activity of 673 genes in L6 cells (20). Hussien and Brooks later showed that both lactate dehydrogenase A (LDHA) and LDHB as well as monocarboxylate transporters (MCTs) were expressed in breast cancer cells, including MCF7 (24).

Most recently, Zhang et al. (25) have shown that “lactylation” of histone lysine residues serves as an epigenetic modification that directly stimulates gene transcription from chromatin in human and mouse cells. They also showed that lysine lactylation (Kla) levels increased in a dose-dependent fashion in response to exogenous L-lactate and that endogenous production of lactate is a key determinant of histone Kla levels (25). Furthermore, Verma's group has recently and elegantly demonstrated that tumor-mediated lactate can elicit epigenomic reprograming of cancer-associated fibroblasts from pancreatic ductal adenocarcinoma (26).

Moreover, there has been growing interest in blocking lactate transport and exchange among and within cancer cells to decrease tumor growth and carcinogenesis (6, 23, 27–29).

Renewed interest in understanding the causes and consequences of the Warburg Effect has shown that lactate can be produced from glutaminolysis. That observation is of consequence because glutaminolysis is considered a hallmark of cancer metabolism (30). It has been known since the 1970's that glutamine is a major energy source for mitochondria in HeLa cells (31) as well as biomass precursor for the proliferation of cancer cells (32). In pediatric glioblastoma cells (SF-188), MYC overexpresses glutaminolysis to elicit a mitochondrial metabolic reprograming favoring glutamine for energy purposes (33). Beyond purposes of bioenergetics and biomass, glutaminolysis can also be a major source of lactate in cancer (34). During high rates of glutaminolysis (a typical characteristic in many cancers) oxidative phosphorylation of glucose is decreased while glutamine fermentation to lactate is increased (34). This concept is important as it could explain why therapies targeting glycolysis may not be very efficient if lactate is derived from glutamine. Furthermore, recent research studies have focused on blocking glutamine metabolism in cancer. In particular one recent study in mouse cancer cells showed that a glutamine agonist JHU082 both shut down oxidative phosphorylation and glycolysis as well as enhanced oxidative phosphorylation and immune response in T-Cells (35).

In breast cancer, there is an average of about 33 somatic mutations (36). Within the multiple somatic mutations in different cancers, there are a few key driver genes that confer a selective growth advantage to “drive” tumorigenesis (36, 37). The driver genes involved in selective growth advantage are referred to as “mut-driver” or “epi-driver” genes (36). Although the epi-driver genes are not yet well-identified or understood, 125 mut-driver genes involved in multiple tumors have been identified (36).

In the present study, we sought to determine whether endogenously produced lactate or exogenously added L-lactate (Sodium L-lactate) exposure could act as an oncometabolite affecting the transcription of key driver genes recognized to play a central role in breast cancer (specifically in MCF7 cells).

## Materials and Methods

### Overview

We tested our lactagenesis hypothesis by exposing MCF7 cells to glucose, which resulted in endogenously produced lactate, or added, exogenous sodium L-lactate and observing the transcription of key driver genes breast cancer, some of which are involved in the majority of cancers (38–44). We took this approach because of the orchestrated action of oncogenes, tumor suppression genes, transcription factors and cell cycle genes that activate an array of pathways leading to increased cell proliferation and the metabolic reprograming of cancer cells from oxidative phosphorylation (OXPHOS) to glycolysis and lactate production.

### Cell Culture Experiments

For both study objectives MCF7 cells (ATCC) were maintained in Dulbecco Modified Eagle Medium (DMEM) with 10% Fetal Bovine Serum (FBS) and Penicillin 100 units/mL-Streptomycin 100 ug/mL (Invitrogen) [DMEM 10% FBS-Pen-Strep] and cultured in a 5% CO_2_ atmosphere at 37°C. Briefly, MCF7 cells (1 × 10^6^) were plated in cell culture dishes using DMEM 10% FBS-PenStrep. Once the cells reached 80% confluence, the cells were incubated in DMEM (high glucose; 4,500 mg/L) containing 0, 10, or 20 mM sodium, L-lactate (Sigma) with 10% NuSerum (BD Biosciences), and Pen-Strep. Controls were MCF7 cells incubated in DMEM without glucose, glutamine, or lactate. Cells were maintained for either 6 or 48 h before being harvested for gene expression profiling. Cell pellets were stored in RLT buffer (Qiagen) with beta mercaptoethanol added, and stored at −80°C. The concentration of lactate and glucose in the cell culture media at the time of harvest was determined using the L-lactate Assay Kit (AbCam) or Glucose Assay Kit (Cayman Chemical), respectively, following manufacturer specifications.

### Cell Line Authentication

MCF7 (ATCC® HTB22™) cells were authenticated by ATCC using morphology, karyotyping, and PCR based approaches to confirm identity of human cells and to rule out both intra- and inter-species contamination. These included an assay to detect species specific variants of the cytochrome C oxidase I gene (COI), and short tandem repeat (STR) profiling. The cell line was obtained in March of 2018 and the last test was done in September of 2018.

### RNA Isolation and Assessment of MCF7 Gene Expression

Total RNA was extracted using the AllPrep DNA/RNA Mini Kit (Qiagen) and the quantity assessed using Nanodrop spectrophotometry. RNA was reverse transcribed using a cDNA conversion kit. The cDNA in combination with RT^2^ SYBR® Green qPCR Mastermix was used on a Custom RT2 Profiler PCR Array (Qiagen). RT2 Profiler PCR Arrays were used to compare gene expression profiles between MCF7 cells cultured in glucose-free media, glucose-supplemented media (leading to Warburg Effect and lactate production) and glucose-supplemented media with exogenous lactate added. Targeted genes typical of MCF-7 cells included on the array were: oncogenes (MYC, NRAS, and PIK3CA), transcription factors (HIF1A and E2F1), tumor suppressor genes (BRCA1 and BRCA2) as well as cell cycle genes, and proliferation genes (AKT1, ATM, CCND1, CDK4, CDKN1A, CDK2b, and MIF) (Table 1). Genes were categorized according to NIH Genetics Home Reference (www.ghr.nlm.nih.gov). Each condition was run in triplicate, and from each of the three biological replicates duplicate samples were run on the expression array to ensure accuracy and reproducibility.

**Table 1:** Fold changes in expression for cancer-related genes between glucose-starved MCF7 cells vs. MCF7 cells exposed to 0, 10, or 20 mM lactate for 6 or 48 h.

**Gene type^+^**	**Incubation duration 6 h**			**Incubation duration 48 h**		
	**6 h, 0 mM**	**6 h, 10 mM**	**6 h, 20 mM**	**48 h, 0 mM**	**48 h, 10 mM**	**48 h, 20 mM**
**PROTO-ONCOGENES**						
NRAS	–	3.56	–	2.31	2.58	1.92
PIK3CA	–	4.31	–	2.04	2.20	2.03
MYC	1.88	7.75	6.28	3.33	2.80	2.81
**CELL CYCLE/PROLIFERATION GENES**						
ATM	–	–	–	8.14	4.04	4.22
CCND1	–	2.60	2.71	2.40	2.33	2.37
CDK4	2.51	6.36	5.64	3.97	3.59	3.02
CDK1A	–	6.77	4.71	2.59	1.36	2.48
CDK2b	1.58	6.82	–	3.75	2.48	2.60
AKT1	1.98	3.35	1.46	2.15	2.72	1.75
MIF	–	7.55	5.19	–	–	2.09
**TUMOR SUPPRESSOR GENES**						
BRCA1	1.70	3.42	3.71	3.42	4.27	2.43
BRCA2	–	6.14	5.55	4.88	4.94	3.31
**TRANSCRIPTION FACTORS**						
HIF1A	–	4.42	4.80	2.87	4.07	2.93
E2F1	1.58	3.37	3.28	2.30	2.56	1.68

*Results are shown for genes showing a 1.5-fold or greater change in expression and a p-value ≤0.05*.

+ *Genes classified according to NIH Genetics Home Reference (www.ghr.nlm.nih.gov)*.

### Statistical Analyses

Lactate and glucose concentrations were compared between groups by analysis of variance (ANOVA) in GraphPad Prism (v. 5.01; GraphPad Software).

Cycle threshold (*C*_*t*_) values, the number of PCR cycles required for florescent signal to exceed background levels, are inversely proportional to the amount of target nucleic acid present in the sample. C_t_ values were exported and then uploaded onto GeneGlobe, Qiagen's data analysis web portal (https://geneglobe.qiagen.com). Within the GeneGlobe platform, Δ*C*_*t*_ values were calculated by subtracting the *C*_*t*_ value for the reference gene (GAPDH) from the *C*_*t*_ value for target genes for each sample. For all experiments, controls were MCF7 cells cultured in glucose/glutamine-free media with 0 mM lactate for 6 h to eliminate the impact of glucose-derived endogenous lactate production (Warburg Effect) on gene expression. Statistical tests were performed on raw Δ*C*_*t*_ values for each group. Fold change was then calculated using 2^−ΔΔCT^ formula. Gene expression differences between experimental groups and controls are expressed as fold-regulation. Criteria for reporting gene expression differences include: fold-regulation of ≥2.0 with a *p*-value of ≤0.05. Lactate and glucose concentrations were compared between groups by analysis of variance (ANOVA) in GraphPad Prism (v. 5.01; GraphPad Software).

## Results

### Glucose-Derived Lactate Production

**Figure 1:**
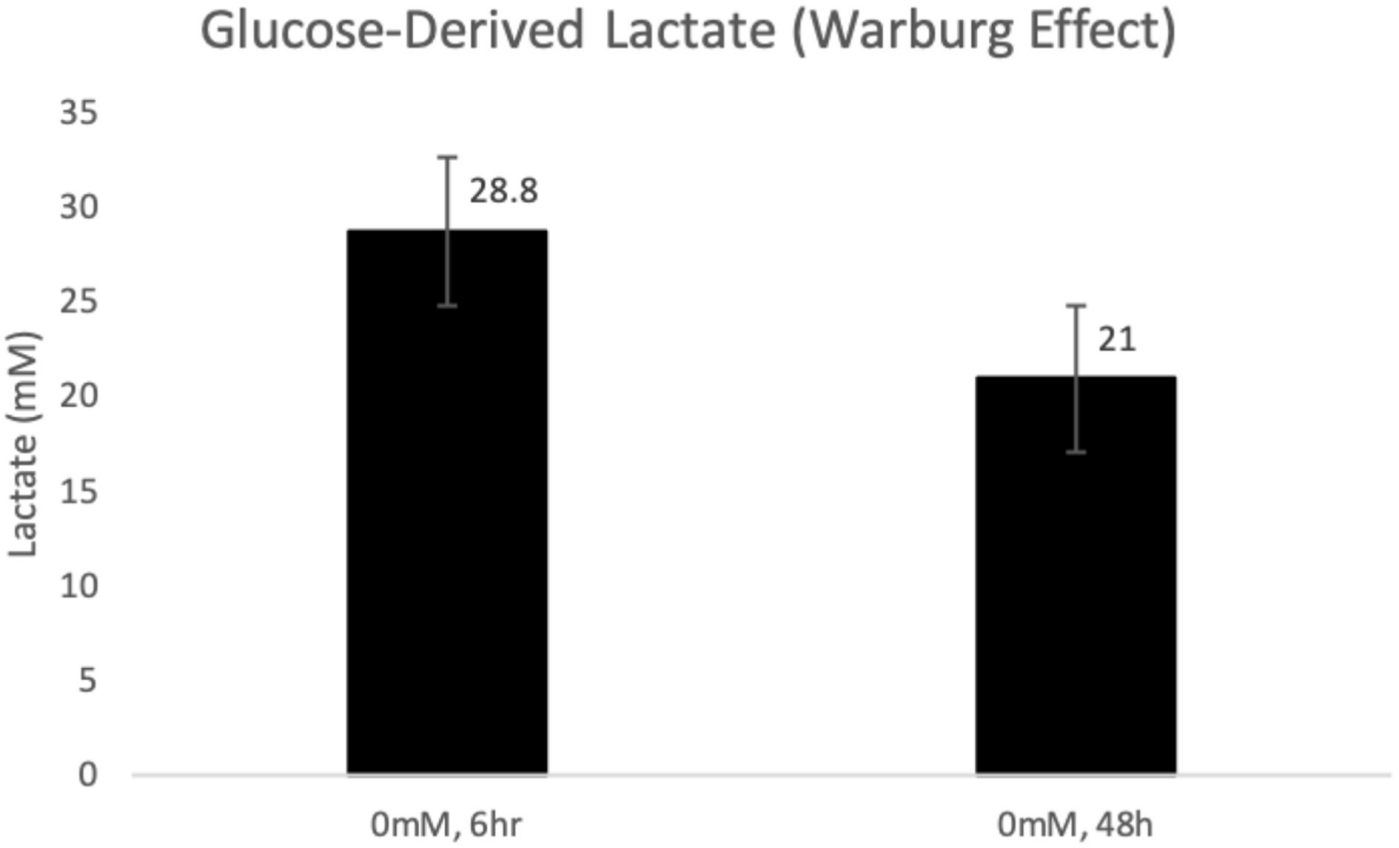
Glucose-derived lactate (Warburg Effect) in MCF-7 cells after 6 and 48 h of incubation in glucose (*p* < 0.001).

As expected, glucose incubation resulted in high concentrations of lactate (28.8 ± 2.9 and 21 ± 6.8, mM (*p* < 0.001) both 6 and 48 h, respectively post-incubation in glucose (Figure 1). As no lactate was added in this experiment in glutamine-free media, lactate accumulation was considered to be glucose-derived as a result of the Warburg Effect.

### Glucose-Derived Endogenous Lactate (Warburg Effect) Upregulates the Transcriptional Activity of Oncogenes, Transcription Factors, Tumor Suppressor Factors as Well as Cell Cycle and Proliferation Genes

**Figure 2:**
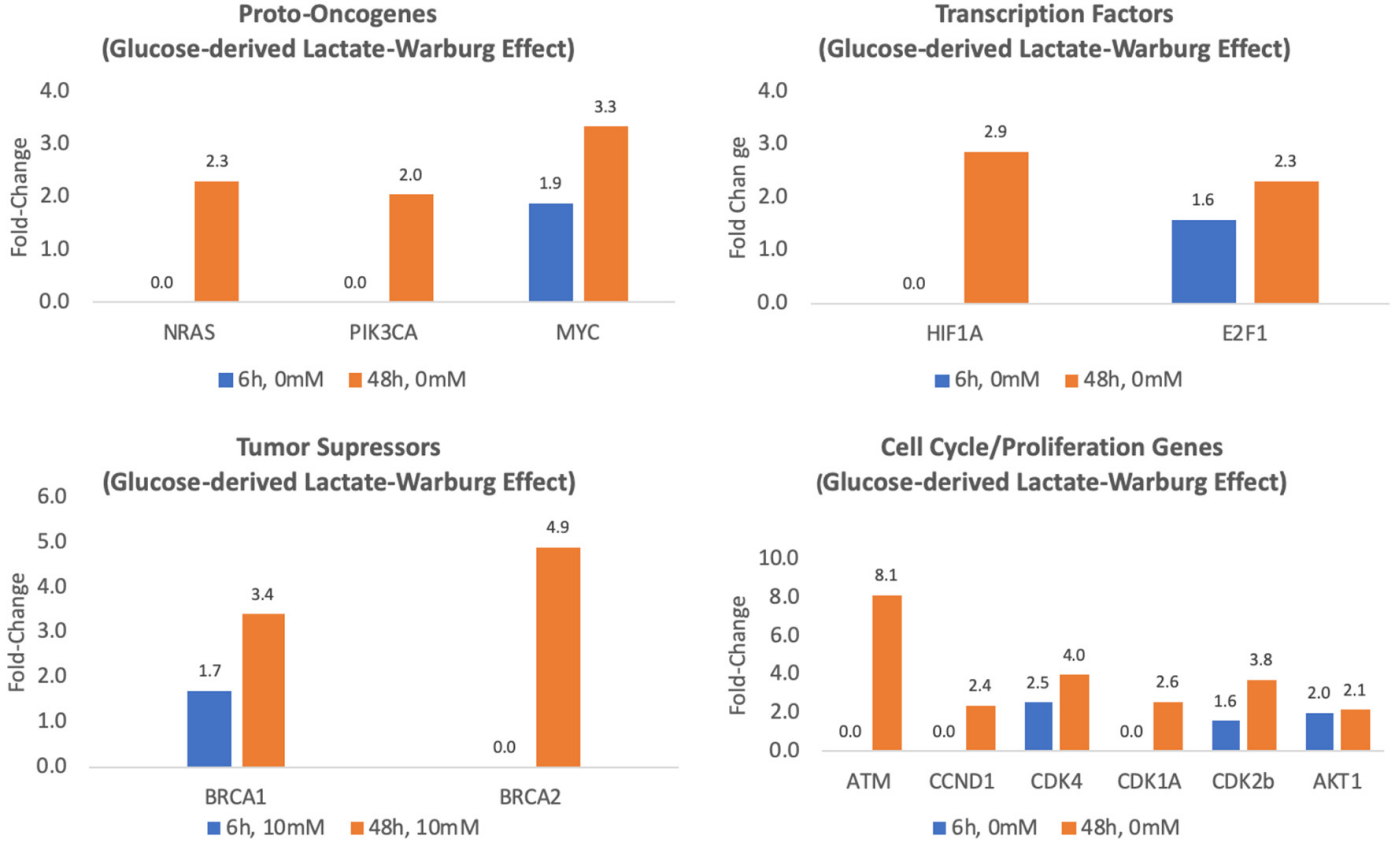
Fold-regulation of Glucose-derived lactate (Warburg Effect) transcriptional activity for MCF7 cells cultured for 6 and 48 h in glucose media relative to controls (MCF7 cells cultured in glucose/glutamine-free media) (*p*'s ≤ 0.05–0.01).

Compared to controls, the expression of three key oncogenes, MYC, RAS, and PIK3CA, was between 2- and 3.3-fold greater in MCF7 cells cultured for 48 h in glucose-containing media leading to lactate accumulation (*p* < 0.05) (Table 1, Figure 2). Endogenous lactate production also affected the expression of transcription factors known to be involved in MCF7 cancer cells. Specifically, compared to controls, MCF7 cells cultured in glucose-containing media for 48 h showed 2.9 and 2.3-fold increases in mRNA expression of transcription factors HIF1A and E2F1, respectively (*p* < 0.01) (Table 1, Figure 2). After 48 h exposure, the transcriptional activity of tumor suppressor factors BRCA1 and BCRA2 increased 3.4- to 4.9-fold, respectively (*p* < 0.01) (Table 1, Figure 2). Finally, transcriptional activities of cell cycle and proliferation genes (except for MIF) increased 2.1- to 8.1-fold (*p* < 0.05) (Table 1, Figure 2) after 48 h exposure between.

### Exogenous Lactate Exposure Upregulates the Transcriptional Activity of Oncogenes, Transcription Factors, Tumor Suppressor Factors as Well as Cell Cycle and Proliferation Genes

#### 10 mM Lactate Exposures

**Figure 3:**
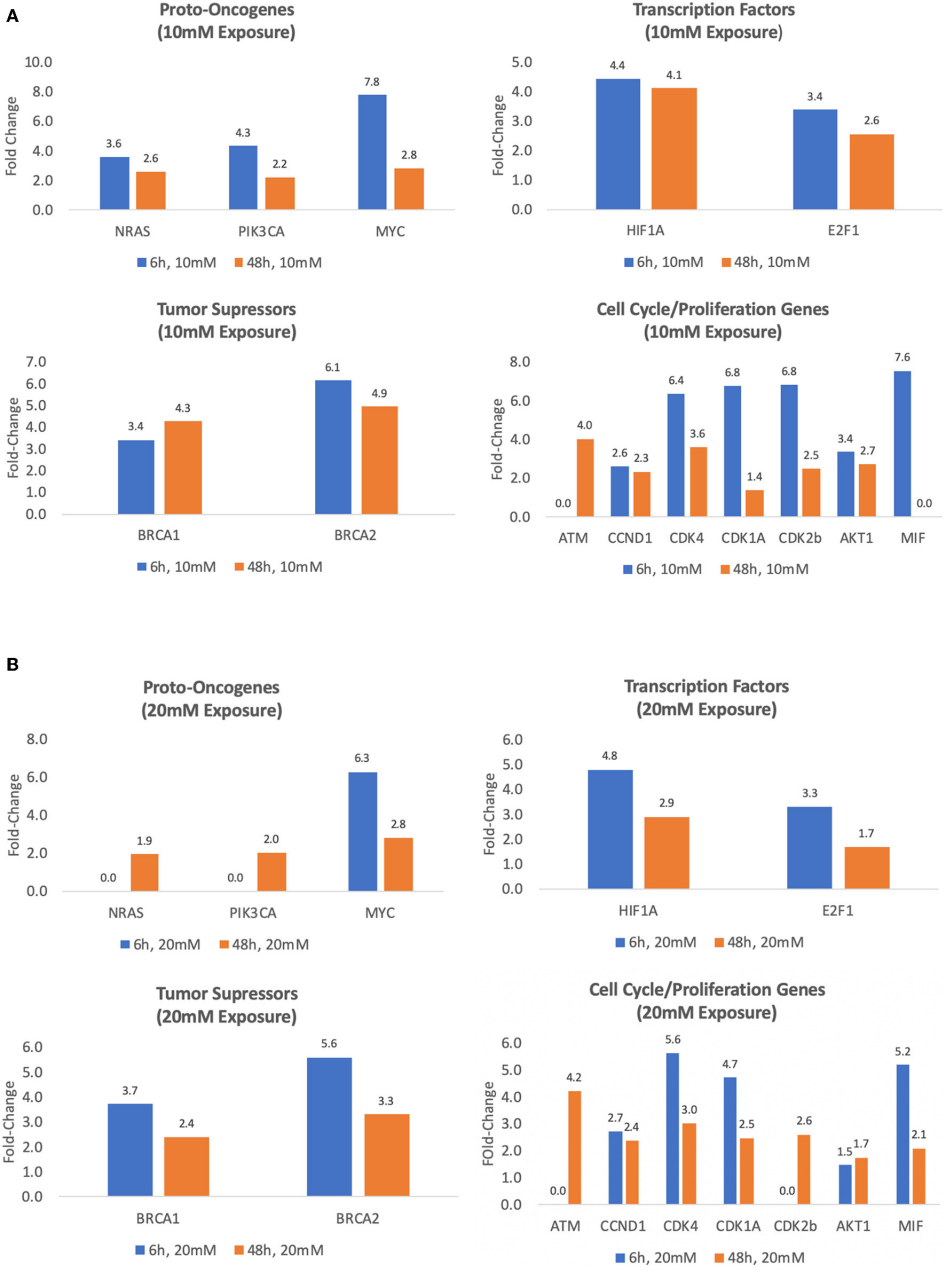
Treatment with exogenous lactate (10 or 20 mM) upregulates the expression of several key genes in MCF7 cells. The fold upregulation of expression with 6 and 48 h exposure to 10 mM **(A)** or 20 mM lactate **(B)** relative to controls (MCF7 cells cultured for 6 h in glucose/glutamine-free media) (*p*'s ≤ 0.05–0.001).

Exposing MCF7 cells to 10 mM lactate for 6 h increased the expression of oncogenes MYC, NRAS, and PIK3CA between 3.6- and 7.8-fold (*p* < 0.05). After 48 h transcriptional activity was slightly weaker for these oncogenes (2.6 and 2.8, *p* < 0.05) (Table 1, Figure 3A). After 6 h, transcription factors, HIF1A and E2F1, were overexpressed by 4.4- and 3.4-fold, respectively (*p* < 0.01) (Table 1, Figure 3A). Expression was similar after the 48 h exposure for HIF1A 4.1-fold, *p* < 0.001), and slightly reduced for E2F1 (2.6-fold, *p* < 0.001) (Table 1, Figure 3A). After 6 h, transcriptional activity of tumor suppressor factors BRCA1 and BRCA2 was increased between 3.4- and 6.1-fold, respectively (*p* < 0.05). After 48 h exposure, transcriptional activities of BCRA1 and BCRA2 increased 4.3- to 4.9-fold, respectively (*p* < 0.001) (Table 1, Figure 3A). Compared to controls, the transcriptional activity of proliferation and cell cycle genes (except for ATM) was significantly greater after 6 h exposure to 10 mM lactate with values ranging from 2.6- to 7.5-fold (all, *p* ≤ 0.05), while a slightly weaker response was observed after the 48 h 10 mM lactate exposure with values (except MIF) ranging from 1.4- to 4-fold (all, *p* ≤ 0.05) (Table 1, Figure 3A).

#### 20 mM Lactate Exposures

In contrast to a 10 mM lactate exposure, neither NRAS nor PIK3CA gene expressions were increased compared to controls in MCF7 cells, while MYC was increased by 6.3-fold (*p* < 0.05) when exposed to 20 mM for 6 h (Table 1, Figure 3B). However, 48 h exposure to 20 mM lactate induced modest increases in NRAS and PIK3CA gene expression (1.9- and 2.0-fold, respectively, *p* < 0.01), and a slight decrease in expression of MYC (2.8-fold, *p* < 0.01) (Table 1, Figure 3B).

Exposure to 20 mM lactate also upregulated the expression of several transcription factors after exposure of 6 or 48 h. At 6 h, gene expression values for HIF1A and E2F1 were 4.8- and 3.3-fold, respectively greater than control (*p* < 0.05) while at 48 h exposure transcriptional activity for HIF1A and E2F1 was slightly lower 2.9- to 1.7-fold, respectively (*p* < 0.05) (Table 1, Figure 3B). Transcriptional activity of tumor suppressor factors after 6 h exposures for BCRA1 and BCRA2 was increased by 3.7- and 5.6-fold, respectively (*p* < 0.001) and was slightly lower after the 48 exposure (2.4- to 3.3-fold, respectively, *p* < 0.001) (Table 1, Figure 3B).

After 6 h exposures, the transcriptional activity of all cell cycle genes except for ATM and CDK2b was overexpressed ranging from 1.5- to 5.6-fold (all, *p* ≤ 0.05) (Table 1, Figure 3B). After 48 h, we observed increased transcriptional activity in all cell cycle and proliferation genes studied ranging from 1.7- to 4.2-fold (*p* < 0.05). Forty-eight hours exposures shown weaker response than at 6 h, but still significant upregulation of cell cycle genes (Table 1, Figure 3B).

## Discussion

Our findings demonstrate that in the MCF7 human breast cancer cell line, lactate alters the transcriptional activity of several key oncogenes as well as other driver genes involved in metabolic reprograming as well as the regulation of cell cycle and proliferation. In the aggregate, these observations are in line with our “lactagenesis hypothesis” (8) positing augmented lactate production for signaling carcinogenesis as one essential purpose of the Warburg Effect.

After both 6 and 48 h exposures there was a high presence of glucose-derived lactate in the cells incubated in glucose without added lactate (or glutamine), which replicated the Warburg studies (Figure 1). Previously, we have shown that lactate is oxidized in mitochondrial preparations from non-transformed tissues (14, 45, 46), and recently it has been confirmed that lactate is also oxidized by mitochondria of cancer cells (6, 24) purportedly for energetics (7, 24). Beyond lactate bioenergetics and biomass properties, our study suggests that glucose-derived lactate is sufficient to alter the transcriptional activity of key oncogenes, transcription factor genes, tumor suppressor genes as well as cell cycle, and proliferation genes, all of which are known to be involved in the development of MCF7 breast cancer cells (Table 1, Figure 2). The experiments, adding 10 and 20 mM of Lactate to MCF7 cells, augmented the transcriptional properties of lactate (Table 1, Figures 3A,B) which supports the hypothesis that lactate could be an oncogenic regulator, an oncometabolite.

Although lactate is the obligatory product of glycolysis under fully aerobic conditions (13), and our findings indicate that the addition of L-lactate to glucose (glutamine-free) media increases the transcriptional activity of the candidate genes studied herein, it is certainly possible that lactate and other metabolites involved in glycolysis, the pentose phosphate pathway or the TCA cycle could also influence the transcriptional activities of various genes in tumorigenesis. For example, Damiani et al. have observed that TCA intermediates that are not used for biomass purposes can be disposed via lactate production (34). Hence, while lactate is a metabolic intermediate, it has numerous downstream effects as known to occur via cell redox changes (14), allosteric binding (47), metabolic reprograming (26), and lactylation (25). The downstream effects of lactate in cancer remain to be determined.

PIK3CA is considered to be the most mutated oncogene in breast cancer (48, 49). Furthermore, PIK3CA mutations are key drivers of breast cancer and its upregulation is associated with poor prognosis (50). Noteworthy, PIK3CA mutations are more frequent in estrogen receptor cancer cells, such as like MCF7 (51). In this study we demonstrate that lactate exposure to MCF7 cells is able to increase the transcriptional activity of PIK3CA between 2.2- and 4.3-fold (*p* < 0.05–0.001) (Table 1, Figures 2, 3A,B). Furthermore, the PIK3/AKT/mTOR pathway is key and important intracellular pathway with major role regulating cell cycle, tumor growth, and proliferation (52, 53), one of the most activated signaling pathways in breast cancer (52) as well as required for survival of MCF7 (54).

In our study, we found that AKT1 transcriptional activity was upregulated between 2- and 3.35-fold. The significant increase in transcriptional activity elicited by lactate in both PIK3CA and AKT1 implicates lactate as a signaling oncometabolite of the key PIK3/AKT/mTOR pathway involved in the development of many cancers.

Another major oncogene, MYC, is known to have multiple roles in metabolic regulation including cellular adaptations following endurance exercise training (55), but is frequently overexpressed in breast cancer cells (56, 57), including MCF7 cells (58), and associated with poor prognosis (57). In our study, we found that MYC is highly expressed across all our experiments between 2.8- and 7.7-fold (*p* < 0.01) (Table 1, Figures 2, 3A,B).

Hypoxia inducible factor 1 (HIF1α) as a major transcription factor in cancer (39, 42). HIF1α increases the transcription of genes regulating glucose transport and glycolytic enzymes (42), eliciting a metabolic reprogramming, leading to the Warburg Effect and lactate production. Furthermore, the overexpression of HIF1A, the gene encoding HIF1α, plays an important role in breast cancer tumor growth and metastasis as well as being related to aggressiveness and poor prognosis (59–61). In all of our lactate exposures experiments HIF1A transcriptional activity was overexpressed (between 2.9- and 4.8-fold, *p* < 0.001) (Table 1, Figures 2, 3A,B), a finding that is not novel, as others have previously found similar results (22). Still, our present results corroborate those of others showing an important effect of lactate on transcriptional activities of this key transcription factor.

Our results showing an effect of lactate on expressions of MYC and HIF1A genes are consistent results of others showing an upregulation of the glycolytic pathway in cancer (62, 63). Hence, our results obtained on transcription of MYC and HIFA are supportive our lactagenesis hypothesis.

BRCA1 and BRCA2 are tumor suppressor genes typically mutated in breast cancer and highly connected with cancer aggressiveness and survival (64–66). BRCA1 contributes to the regulation of DNA repair, chromosomal remodeling, apoptosis, cell-cycle control, and transcriptional activity (67). While the loss or reduced expression of nuclear BRCA1 is prevalent in basal-like breast cancers with negative estrogen, progesterone, and epidermal growth factor receptors (triple negative), its cytosolic expression is observed in estrogen-positive receptor breast cancers (68). In estrogen-positive receptor breast cancer cells (the characteristic of MCF7 cells), cytosolic BRCA1 expression is inversely related to survival (68). Furthermore, transcriptional activity of BRCA1 and BRCA2 genes has been observed in multiple breast cancers (including MCF-7 cells) (69–71). In our study, we found that lactate exposure is a potent regulator of their transcriptional activity with increases in mRNA expression between 3.3- and 6.1-fold (*p* < 0.001) (Table 1, Figures 2, 3A,B).

Increased cell cycle and proliferation is a characteristic of cancer cells where all different phases in cell cycle are affected in cancer mainly by cyclin-dependent kinases (CDKs) (72). Among the significant results, we found that all CDKs were overexpressed by lactate exposure in a range from 2- to 6.7-fold (*p* < 0.01–0.05) (Table 1, Figures 2, 3A,B). While the trigger of this genetic dysregulation hasn't been elucidated, our data show that most genes involved in the different phases of cell cycle are overexpressed by glucose-derived lactate alone as well as exogenous lactate; again, implicating lactate as a regulator of CDKs, thus shedding new possible light in cancer cell division and proliferation as well as therapeutics.

The traditional view of dysregulated downstream signaling pathways in cancer is hierarchically mediated by somatic mutations mainly due to dysregulation of oncogenes and tumor suppressors (73, 74). Our results show that, at least in MCF7 cells, lactate doesn't obey a hierarchical order of signaling, and also that in MCF7 cells, lactate signals multiple key steps essential in carcinogenesis, including cell proliferation.

It has been estimated that each gene driver mutation confers only a small selective growth advantage, about 0.4% increase in the difference between cell birth and death (75). However, this small difference over many years can result in significant production and accumulation of tumor cells leading to cancer (36). Likewise, we believe that a similar phenomenon can hold true for the constant transcriptional activity elicited by dysregulated lactate on the main key driver genes over the years.

Furthermore, Marticorena et al. (76) have recently shown that genetic mutation alone could not be a necessary element for cancer development as in their study, they found that both non-cancerous and cancerous esophagus cells shared cancer-associated genetic mutations.

Beyond the roles of oncogenes and tumor suppressor factors, others have speculated that Epi-drivers, like epigenetic changes affecting DNA and chromatin proteins could also be involved in carcinogenesis (36). As mentioned above, a remarkable new study by Zhang et al. has shown the regulation of gene expression by histone “lactylation,” where both exogenous and endogenous lactate levels stimulate gene transcription from chromatin in human and mouse (25). From the extensive work of others, it is known that many mechanisms are also involved in histone acetylation in cancer. A classic example is the retinoblastoma pathway. Once hypophosphorylated at the beginning of G1 phase, retinoblastoma protein (pRb) is hyperphosphorilated at the end of G1 phase and the E2F1/pRB complex breaks off, allowing transcriptional activity of E2F1 at the end of G1 phase. E2F1 can then recruit histone acetylase for acetylation allowing chromatin transcription of genes to facilitate cell cycle moving passed the restriction (R) point into the G1/S transition and S-phase of cell cycle. In our study we show that lactate increases E2F1 mRNA transcription between 1.6- and 4.1-fold (*p* < 0.05–0.001). Again, results support our lactagenesis hypothesis.

In concert, here we show that at least in MCF7 cells, lactate acts as an oncometabolite capable of regulating transcriptional activities of key oncogenes, transcription factors, tumor suppressors, and cell cycle genes involved in breast cancer.

An imperative question we pose now is what cell-specific properties, and mechanisms allow lactate to induce candidate cells toward a cancer phenotype. We have a plethora of knowledge and expertise about muscle (77, 78) and whole-body lactate metabolism during exercise (14) and as mentioned *vide supra*, we have known for decades that lactate is a major source of cellular energy, especially for mitochondria. In normal physiology, there is a dynamic, order of magnitude, range of muscle lactate production, and accumulation (14). However, as a tissue, muscle is resistant to carcinogenesis. In fact, rhabdomyosarcoma, historically thought to be a rare form of muscle cancer, has been recently proven to raise from endothelial progenitor cells following metabolic reprogramming and myogenic transdifferentiation, but not being originated from myocytes in the tissue itself (79). As well, from epidemiology, we know that regular exercise reduces the incidences of some forms of cancers in addition to other chronic diseases (80). Although lactate has been historically associated to exercise, it is noteworthy to differentiate between effects of transient increases in exercise-derived lactate and chronic lactate elevation in cancer. During and after exercise, lactate is ultimately cleared from muscle fibers with the clearance rate depending on mitochondrial function and cardiometabolic fitness level of the person. In contrast, in cancer, lactate is not rapidly cleared, and is highly concentrated in the tumor and its microenvironment; an effect of which could be to promote carcinogenesis.

### Study Limitations

We acknowledge that the present exploratory study has been conducted on a cancer cell line (MCF7). Hence, for further testing of the lactagenesis hypothesis will be important to reproduce this study with other cancer cell lines as well as with tumor biopsy cultures to show generality of the findings and reveal the mechanisms by which dysregulated lactate metabolism could act as an oncometabolite in carcinogenesis.

As well, we acknowledge that effects of treatment on gene transcription are not perfect predictors of protein synthesis and circulating protein levels. However, mRNA levels do show a positive correlation with protein expression (81) with a significant amount of protein (40%) being correlated to mRNA levels (82). Likewise, protein expression alone is a perfect predictor of the ultimate biological action, as the completion of a biological action is due to a compendium of multiple epigenetic effectors including the tumor microenvironment in the case of cancer. In this exploratory study, our objective was to determine the impact of lactate exposure on the expression of key genes known to be involved in the pathogenesis of cancer in MCF7 cells. Future work will be expanded to include the assessment of protein levels for differentially expressed genes.

In summary, our study supports the hypothesis that lactate has the potential to serve as an oncometabolite, regulating transcriptional activities of different key cancer-related genes involved in metabolic reprograming as well as cell cycle and proliferation (*p*'s < 0.05–0.001). Beyond present results with MCF-7 cells additional studies on different cancer cell lines and cultured tumor biopsy cells will be needed to further support the lactagenesis hypothesis and to better understand the role of lactate in carcinogenesis.

## Data Availability Statement

The raw data supporting the conclusions of this manuscript will be made available by the authors, without undue reservation, to any qualified researcher.

## Author Contributions

IS-M and GB contributed to the hypothesis and experiments design as well as the preparation of the manuscript. CJ contributed to the experiments and also to the manuscript. CM contributed to the experiments. JM, IS-M, and GB contributed to the preparation of the manuscript.

### Conflict of Interest

The authors declare that the research was conducted in the absence of any commercial or financial relationships that could be construed as a potential conflict of interest.
